# Multi-Set Testing Strategies Show Good Behavior When Applied to Very Large Sets of Rare Variants

**DOI:** 10.3389/fgene.2020.591606

**Published:** 2020-11-09

**Authors:** Ruby Fore, Jaden Boehme, Kevin Li, Jason Westra, Nathan Tintle

**Affiliations:** ^1^Department of Biostatistics, Brown University, Providence, RI, United States; ^2^Department of Mathematics, Oregon State University, Corvallis, OR, United States; ^3^Department of Mathematics, School of Arts and Sciences, Columbia University, New York, NY, United States; ^4^Department of Mathematics and Statistics, Dordt University, Sioux Center, IA, United States

**Keywords:** rare variant analysis, statistical genetics, missing heritability, power investigation, pathway testing

## Abstract

Gene-based tests of association (e.g., variance components and burden tests) are now common practice for analyses attempting to elucidate the contribution of rare genetic variants on common disease. As sequencing datasets continue to grow in size, the number of variants within each set (e.g., gene) being tested is also continuing to grow. Pathway-based methods have been used to allow for the initial aggregation of gene-based statistical evidence and then the subsequent aggregation of evidence across the pathway. This “multi-set” approach (first gene-based test, followed by pathway-based) lacks thorough exploration in regard to evaluating genotype–phenotype associations in the age of large, sequenced datasets. In particular, we wonder whether there are statistical and biological characteristics that make the multi-set approach optimal vs. simply doing all gene-based tests? In this paper, we provide an intuitive framework for evaluating these questions and use simulated data to affirm us this intuition. A real data application is provided demonstrating how our insights manifest themselves in practice. Ultimately, we find that when initial subsets are biologically informative (e.g., tending to aggregate causal genetic variants within one or more subsets, often genes), multi-set strategies can improve statistical power, with particular gains in cases where causal variants are aggregated in subsets with less variants overall (high proportion of causal variants in the subset). However, we find that there is little advantage when the sets are non-informative (similar proportion of causal variants in the subsets). Our application to real data further demonstrates this intuition. In practice, we recommend wider use of pathway-based methods and further exploration of optimal ways of aggregating variants into subsets based on emerging biological evidence of the genetic architecture of complex disease.

## Introduction

With continued dramatic growth in the amount of sequencing data available, there is a persistent interest in exploring the role that common and rare genetic variants may have in explaining the etiology of complex phenotypes. In particular, there is continued interest in the potential role of rare variation in explaining some of the “missing heritability” (Manolio et al., 2009) for complex phenotypes given that identified causal common variants still account for little of the total observed heritable phenotypic variation for many traits. For example, rare variants are thought to directly contribute to common diseases such as diabetes, schizophrenia, and heart disease (Cirulli and Goldstein, 2010; Bomba et al., 2017).

Within the last decade, numerous methods of summarizing the relationships between sets of rare (and/or common) genetic variants have been proposed. The most common and widely used approaches conduct a single test of the null hypothesis that none of the single-nucleotide variants within a set of interest (typically a gene) are associated with the phenotype of interest. There are two broad classes of such tests: burden tests [e.g., CMC (Li and Leal, 2008)] and variance-component tests [e.g., SKAT (Wu et al., 2011)], with the main distinction being whether or not the test accounts for the potential beneficial (protective) impact of rare variants on disease, which is the case for variance-component tests, but not burden tests. In general terms, burden tests may be more powerful when risk-impacting variants have generally similar effects, whereas variance-component tests may be more powerful when there is heterogeneity in the variant effects (Basu and Pan, 2011; Liu et al., 2013), with a new class of approaches attempting to optimally combine these two broad classes (Lee et al., 2012; Greco et al., 2016), though simulation evidence suggests that no one method is universally most powerful (Ladouceur et al., 2012; Derkach et al., 2014).

As the amount and accessibility of sequence data has continued to increase [e.g., the UK Biobank (Sudlow et al., 2015)], gene-based tests of rare variants (both burden and variance-components approaches) are being used with larger and larger numbers of variants within the sets. While the computational hurdles associated with testing very large sets (e.g., 100s to 1000s of variants) simultaneously are being addressed with ongoing research (Lumley et al., 2018), less is known about the statistical performance of burden and variance-component tests in these situations.

For almost two decades, the class of pathway analysis methods [e.g., GSEA; (Subramanian et al., 2005)] have been proposed as methods for aggregating gene-based summary statistics across multiple genes within a biologically defined set (e.g., a pathway). Although many of the original pathway analysis methods were developed for use on gene-expression data, the application of these methods to sequencing data has been proposed (Wu et al., 2010; Wu and Zhi, 2013) and applied specifically to analyze rare variants (Aslibekyan et al., 2014; Moore et al., 2016; Richardson et al., 2016; Larson et al., 2017). These approaches first conduct gene-based tests of association and then use methods to aggregate the gene-level test statistics (Petersen et al., 2011; Aslibekyan et al., 2014; Valcarcel et al., 2016). While these preliminary analyses have demonstrated that pathway analysis is a valid approach for sequencing data, there has been little systematic evaluation of the performance of pathway-based methods in conjunction with gene-based tests (burden and variance-components). A crucial question is whether, for a given set of single-nucleotide variants (SNVs) from a functional pathway, a pathway-based strategy may outperform a gene-based testing strategy with a multiple testing correction, which is currently seen in the literature (Neuhaus and Kalbfleisch, 2006; Kim et al., 2017; Oh et al., 2019; Shivakumar et al., 2019). Importantly, as the numbers of variants within gene-based “sets” continue to increase, a multi-set testing strategy may become more theoretically and computationally appealing.

More generally, a gene-based testing strategy can be thought of as a *single-set testing* strategy—the evidence of association for each variant in the set of interest with the phenotype can be summarized with a single value (e.g., the *p*-value from SKAT applied to all rare variants within a gene of interest). Alternatively, pathway methods can be thought of as a *multi-set testing* strategy—first, the variants in the set of interest are grouped into mutually exclusive subsets, evidence of phenotypic association is aggregated for each subset, and finally, the evidence for each subset is aggregated to a single test statistic for the entire set. While historically the subsetting process involved genes and a secondary aggregation step involved pathways (sets of genes with biologically related function), other subsetting approaches are possible. For example, the subsets could involve each exon/intron in the single-set test and the gene at the aggregation level, with numerous other alternative grouping strategies possible (Moore et al., 2013). Thus, the conceptual contrast is between a single-set testing strategy (all variants in one single set) and a multi-set testing strategy (variants are first grouped, evidence of phenotype association is evaluated for each group, and then the evidence is aggregated across the groups).

In this paper, we will provide a general framework for exploring single-set vs. multi-set testing strategies for tests on sets of rare variants. Simulation will be used to explore our proposed testing strategy in several distinct hypothetical scenarios, and we demonstrate the utility of our approach in an application to a real dataset. Our goal is to evaluate under which conditions single-set vs. multi-set testing strategies may be statistically advantageous. Ultimately, we will make practical suggestions for researchers considering testing sets of 100s to 1000s of genetic variants.

## Materials and Methods

### Terminology

The multi-set (alternatively called, “pathway-based”) approach we explore here requires a test statistic that aggregates information at the single-set (or gene) level, and a secondary aggregation statistic that aggregates multiple, single-set statistics at the “pathway” level. While we use the terms “gene” and “pathway” here, it is important to note that our investigation is focused on the broader question of whether or not to conduct a single test of all variants of interest (single-set or gene-based only strategy) or a multi-set strategy (subsets of all variants tested individually first with a single-set strategy, and then aggregated using a pathway based statistic).

We note that the historically used terminology “pathway-based method” and “gene-based method” is somewhat limiting. “Gene-based” tests can be conducted on any set of variants, for example, individual exons, introns, windows of the genome, regulatory binding sites, etc., Similarly, “pathway-based” methods do not need to be run at the pathway level (set of biologically related genes). More broadly, “pathway-based” methods can be conducted to aggregate any set of individual set-based statistics, including *p*-values. For example, a “pathway-based” method could be used to aggregate all of the intron and exon level set-statistics. To support these broader definition and interpretation, we will use the terms “multi-set” and “single set” throughout the remainder of the paper.

### Implementation of the Single-Set Method Using the Sequence Kernel Association Test

As the primary test of association between genetic data and phenotypes, we will explore a generic version of a variance component test of association. We use a mathematically equivalent version of the Sequence Kernel Association Test (SKAT) at the subset (or “gene”) level, which allows for more straightforward exploration of the behaviors of the SKAT/variance components statistic (Liu et al., 2013). We define our variance component (VC) test statistic as follows:

$$\text{VC}=∥{\text{F}}^{+}-{\text{F}}^{-}∥$$

where *F* ^+^ is th e vector of allele frequencies in the set of variants of interest for the cases, and *F*^−^ is the same quantity for controls. For an individual rare variant *h* = 1,2…,*l*, an element of *F*^+^ is defined as the count of rare alleles among the cases divided by the number of possible alleles in the cases,  2*N*_A_, where *N*_A_ is the number of cases. Thus, for a matrix of *l* SNVs for *N* individuals, ∥*F*^+^-*F*^−^∥ tests the null hypothesis of no difference in the allele frequencies between the cases and controls for the *l* variants in the set.

In addition to the generic variance component test, we also explored the behavior of a generic burden test and saw a similar behavior to what we observe in Section 3 (detailed results for burden test not shown).

### Implementation of the Multi-Set Method

#### Multi-Set Testing Strategy

Like the single-set testing strategy, the multi-set testing strategy evaluates a matrix of rare allele counts over *l* SNV sites and *N* individuals. However, the test also requires a division of variants into *m* mutually exclusive and exhaustive subsets of variants.

The testing strategy proceeds as follows:

1) Break the SNV data into *m* mutually exclusive and exhaustive subsets
2) Calculate the single-set statistic *t* on each of the *m* subsets so that we have *t*_1_,*t*_2_…*t*_*m*_. In this case, we use the SKAT-equivalent statistic described in 2.2. Combine test statistics *t*_1_,*t*_2_…*t*_*m*_ using an aggregating statistic, *T* (see section “Choices of Aggregating Statistics”).
3) Permute case–control status *P* times and recalculate single-set statistics on each set, to obtain test statistics of the randomly permuted data *s*_*k*1_,*s*_*k*2_…*s*_*km*_ for permutation *k* ∈ {1,2,…*P*}. Then, compute the aggregating statistic on the single-set statistics computed on the permuted data, *T*_*k*_.
4) Compute the *p*-value for the aggregating statistic by comparing *T* to *T_1_*,…,*T_*P*_.*

#### Choices of Aggregating Statistics

We consider three different choices of aggregating statistics.

##### Fisher’s method

Fisher’s method combines*m p*-values into a test statistic with a $\chi_{2\,m}^{2}$ distribution with the following formula: T = $-2\,\sum_{i=1}^{\text{m}}\text{ln}\,\left(p_{i}\right)$, where *p*_*i*_ is the *p*-value corresponding to the single-set statistic *t*_*i*_, computed on the *i*^th^ subset of variants.

##### Sumstat

SUMSTAT is defined as $T=\sum_{1}^{m}t_{j}$ (Tintle et al., 2009a,b), where *t*_*i*_ is the single-set statistic computed on the *i*^*th*^ subset of variants.

##### Bonferroni correction

For a set of *m p*-values, *p*_1_,*p*_2_,…*p*_*m*_, *T* = Min(p) = *min*(*p*_1_,*p*_2_,…*p*_*m*_), where, *p*_*i*_ is the *p*-value corresponding to the single-set statistic *t*_*i*_, computed on the *i*^*th*^ subset of variants and the aggregating test is considered statistically significant if Min(p) is less than 0.05/*m*.

We consider the aggregation methods in relation to each other as well as in comparison to testing all the causal variants in a single set, without sub-setting variants (the “gene-based” method).

### Simulations

We investigated the power of our proposed testing strategy in several related scenarios. In all scenarios, we considered a total of *l* = 1024 SNVs, and *N* = 1000 individuals, evenly split into 500 “cases” and 500 “controls.” In all scenarios, 128 of the total 1024 SNVs (12.5%) were causal with risk ratio of 1.15, while the remaining 896 SNVs were non-causal (risk ratio of 1).

We considered four separate simulation scenarios. The two primary attributes varied in our simulations were (a) equal (or unequal) numbers of variants in each subset and (b) equal (or unequal) proportions of causal variants in each of the subsets. We looked at four primary simulation scenarios: one for each of the combinations of equal/unequal numbers of variants and equal/unequal proportions of causal variants (2 × 2 factorial design). A summary of simulation scenarios is provided in Table 1.

**TABLE 1:** Summary of simulation scenarios investigated.

	**Proportion of causal variants per set is the same in all sets**	**Proportion of causal variants per set differs across sets**
Equal numbers of variants per set	*Scenario #1*	*Scenario #3*
Unequal numbers of variants per set	*Scenario #2*	*Scenario #4*

Specifically, in *Scenario #1*, we considered different values of *m* (the number of subsets), ranging from 1 to 128 (8 to 1024 variants per subset), while maintaining the same proportion of causal variants (1/8) in each subset and equally distributing the variants across each of the *m* subsets. In *Scenario #2*, we fixed the proportion of causal variants at 1/8 in each subset, set *m* = 2, and then explored different set sizes for the two subsets, including set sizes of 8, 16, 32, 64, 128, 256, and 512 SNVs in the smaller set, with all remaining variants in the other set. *Scenario #3* was similar to *Scenario #1* in that we varied the number of subsets, *m*, and had equal numbers of variants in each subset, but we varied the distribution of the causal variants so that 1/8, 1/4, 1/2, or 3/4 of the total sets contained 100% of the causal variants. Finally, in *Scenario #4* we considered both unequal set sizes and unequal proportions of causal variants across the sets, a more realistic scenario. In particular, we considered divisions with *m* = 2 and 128, 356, 384, or 512 variants in the smaller subset and then varied the distribution of causal variants so that the smaller set contained none, some, or all of the 128 causal variants, with the proportion causal dependent on the size of the smaller set. For a single power estimate, we performed 1000 simulations for that combination of parameters to yield a max margin of error of 3.5% (power = 50%) and margin of error of 1.4% when the type I error rate is 0.05. Power was calculated as the percent of simulations significant at the 0.05 alpha level.

Prior to running simulations for our factorial design, we evaluated the type I error rate of our method by setting the risk ratios for all SNVs to one. The resulting estimate of type I error for SUMSTAT had a range of 3.8%–5.4%, while for Fisher’s it was 4.0%–5.7%, showing empirical control of the type I error rate at the nominal level of 0.05.

### Application to Real Data

To investigate the performance of the multiset testing approach on real data, we used imputed genetic data from 1856 participants of the Framingham Heart Study Offspring cohort (dbGaP Study Accession: phs000007.v29.p10). We extracted SNPs from the free fatty-acid receptor (FFAR) family of genes (FFAR1–FFAR4), the fatty-acid desaturase family (FADS1–3), and the elongation of very long chain protein 2 (ELOVL2) gene. One of the genes in the free fatty-acid receptor family, FFAR4, has a protein product (G-protein coupled receptor 120) known to have an effect on the metabolism and tasting of fats (Cartoni et al., 2010; Song et al., 2017), and so we utilized our method to test rare SNVs in this collection of genes for their association with obesity [dichotomized as obese (BMI greater than 30 kg/m^2^) vs. not obese (BMI less than or equal to 30 kg/m^2^)] in the cohort. We tested for association with obesity category on two different gene sets: FFAR genes alone, and the entire set. Genetic data was filtered after imputation for a MAF < 0.05, imputation *R*^2^ greater than 0.4, and imputed dosages in the ranges (0.2, 0.8) and (1.2, 1.8) were called missing while the remaining genotype dosages were rounded to the nearest whole number. The remaining SNVs were separated into sets defined by gene, and this division of the total SNVs resulted in decidedly unequal set sizes (Table 2), ranging from 15 SNVs in FFAR1 and FFAR3 to 322 SNVs in ELOVL2, a more than 20-fold difference in set size. Given the existing evidence of the impact of FFAR4 on obesity, we also hypothesized that the proportion of causal variants would be unequal between subsets. Unequal set sizes and unequal distribution of causal variants among sets are extremely plausible in the analysis of real data, and while the true distribution of causal variants in our entire set of SNVs is unknown, we certainly see unequal set sizes in our real data application.

**TABLE 2:** *P*-values produced by multi-set testing as compared to single-set testing.

	**Multi-set *p*-value (Fisher’s)**	**Multi-set *p*-value (SUMSTAT)**	**Multi-set Bonferroni *p*-value^1^**	**Single-set *p*-value**
FFAR gene family (4 genes)	0.051	***0.037***	0.019	0.064
Fatty acid gene family (8 genes)	***0.017***	***0.033***	0.013	0.05

*^1^Emphasized *p*-values are those that reach their respective significance levels. For Bonferroni, the *p*-value illustrated is the smallest *p*-value across the genes tested. The significance level must be adjusted to 0.05/4 = 0.0125 and 0.05/8 = 0.00625 for the two testing scenarios, respectively, meaning that even though the illustrated *p*-value is the smallest, this finding would not hold up to multiple testing penalties. Bold here denotes reaching statistical significance.*

## Results

### Equal Proportions of Causal Variants Across Subsets (Scenarios #1 and #2)

**FIGURE 1:**
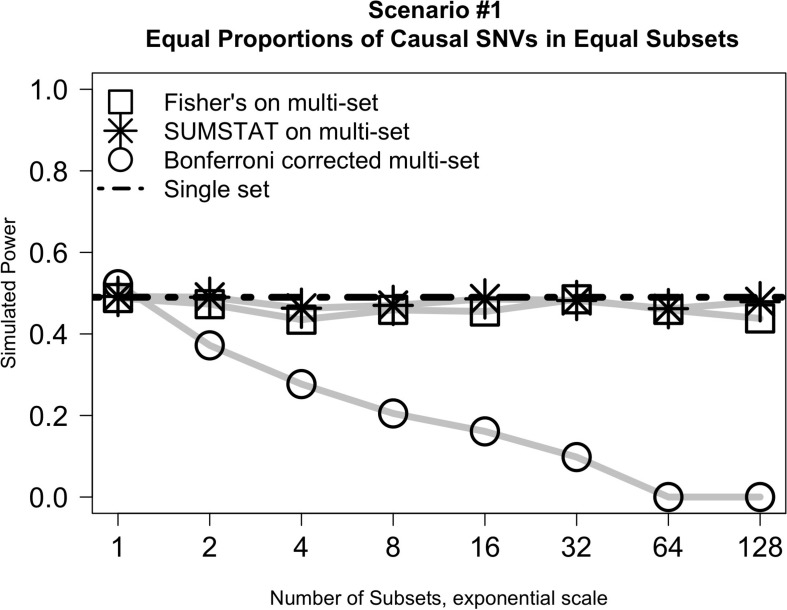
Multi-set approaches using aggregation statistics have improved power over multi-set testing with a Bonferroni correction as the number of subsets increases.

Results from simulation *Scenario #1* are illustrated in Figure 1. In short, when subsets are equally sized and the proportion of causal variants in each subset is held constant, the power of the Fisher’s and SUMSTAT multi-set approaches are similar, though slightly lower, than the power of a single-set approach. Importantly, this holds true even as the number of subsets increases. However, multi-set testing using a Bonferroni correction for multiple testing loses power rapidly as the number of sets increases.

**FIGURE 2:**
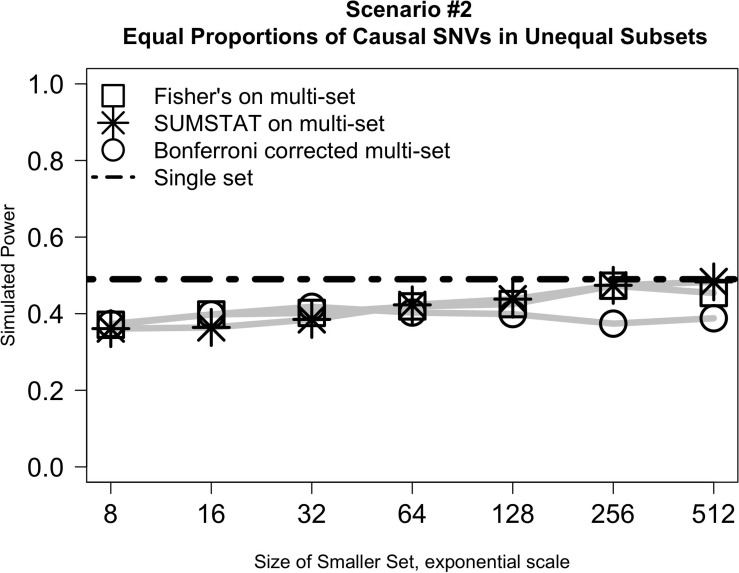
Relative subset size has little impact on the power of multi-set approaches when the proportion of causal variants is equally distributed between two sets.

Figure 2 illustrates the results from *Scenario #2*, which explored test behavior when the proportion of causal variants was the same in both subsets, but subsets were of different sizes. In this case, the difference in size between the two subsets had little impact on power even as the ratio of set sizes ranged from 1:128 to 1:1. SUMSTAT and Fisher’s both gained some power as set sizes became more equal, and Bonferroni lost power slightly, but the overall change was small. Notably, however, power was less than a single-set approach in all cases. The decrease in power as compared to a single set could be explained by considering the aggregating methods to be weighting the two sets equally in creating the final *p*-value when in fact their size and number of causal variants are unequal. When variants are distributed equally, dividing into unequal subsets results in down weighting the signal of the causal variants in the larger subset.

### Unequal Proportions of Causal Variants Across Subsets (Scenarios #3 and #4)

**FIGURE 3:**
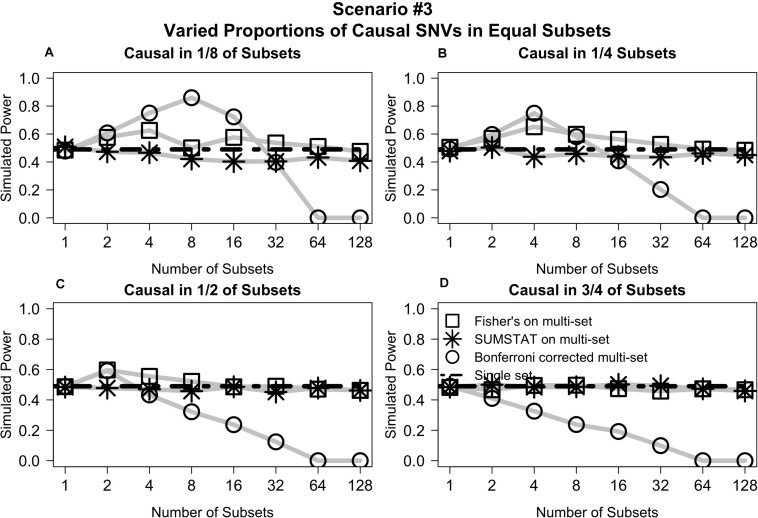
Multi-set aggregation statistics Fisher’s and SUMSTAT yielded power similar to a single set test, whereas Bonferroni showed generally worse power for large number of subsets, with some specific cases where Bonferroni improved power. **(A)** Power curves when subsets are equally sized and causal variants are distributed among only 1/8 of the total subsets. **(B)** When casual variants are distributed among 1/4 of the subsets. **(C)** When causal variants are distributed among 1/2 of the subsets. **(D)** When causal variants are distributed among 3/4 of the subsets.

Varying the distribution of causal variants but maintaining equal set sizes in *Scenario #3* shows that the Fisher’s and SUMSTAT multi-set and single-set approaches maintain steady power curves (Figure 3). In general, the Bonferroni approach yields low power for large numbers of sets. However, in a few unique cases the Bonferroni approach yields better power than other methods. When causal SNVs are aggregated together, the power of Bonferroni peaks at the set division that corresponds to testing all causal variants in one single set. Since Bonferroni uses the minimum *p*-value across the sets as the primary test statistic, this scenario maximizes power.

**FIGURE 4:**
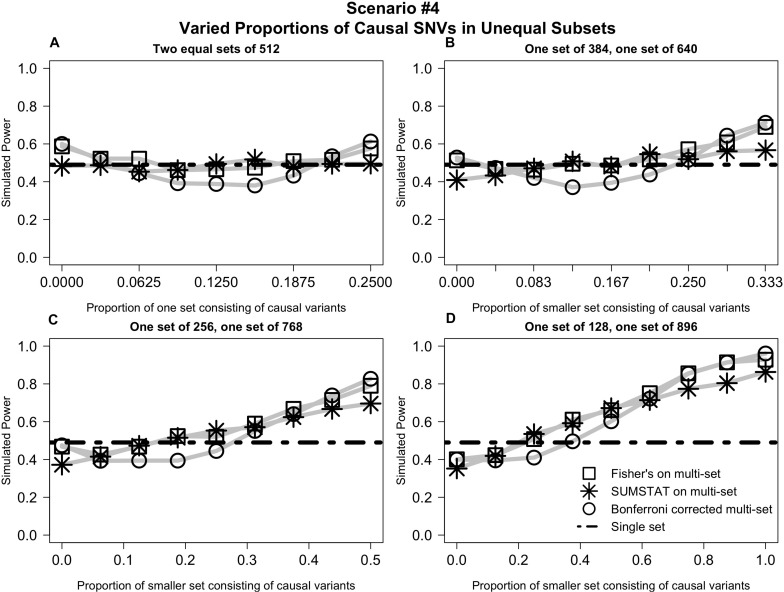
Varied distribution of causal variants into unequal subsets demonstrates increased power for multi-set methods as the proportion of causal variants increases in the smaller set. **(A)** Power curves when the SNVs are divided into two equal subsets, and the proportion causal in one subset is varied from 0 to 1/4. **(B)** Power curves when SNVs are divided into two unequal subsets, and the proportion causal in the smaller set is varied from 0 to 1/3. **(C)** Power curves when SNVs are divided into two subsets, one three times the size of the other, and the proportion causal in the smaller set is varied from 0 to 1/2. **(D)** Power curves when SNVs are divided into two subsets, one seven times the size of the other, and proportion causal in the smaller set is varied from 0 to 1.

In *Scenario #4* (depicted in Figure 4), distributing causal variants between two equal sets had no impact on power (4a). However, as we moved non-causal SNVs to the larger set and causal SNVs to the smaller set, power increased for all multi-set methods (Figures 4B–D).

### Results of Application to Real Data

The results of applying a multi-set approach to rare variants in the fatty acid gene family are presented in Table 2, contrasting with a Bonferroni adjustment and a single-set approach. Bold and italicized *p*-values are those that reach the alpha < 0.05 significance level. While the Bonferroni *p*-values are the smallest overall, they do not reach their respective cutoffs for significance after accounting for multiple testing. Out of the four approaches we investigated through simulation (Section “Choices of Aggregating Statistics”) only the multi-set approaches (Fisher’s and SUMSTAT) identified an association between rare variants in the sets of fatty acid genes and obesity.

While there is preexisting knowledge surrounding the FFAR4 gene’s association with fat tasting and metabolism, the smaller *p*-value for Fisher’s method when including the whole pathway as compared to the FFAR family alone indicates possible associations outside the FFAR family. Given that Fisher’s is a *p*-value-based aggregation method, a natural “*post hoc*” analysis after determining significance at the pathway level is to look at the *p*-values from each gene (Table 3).

**TABLE 3:** Rare variant set sizes in application to real data.

**Gene name**	**FFAR1**	**FFAR2**	**FFAR3**	**FFAR4**	**FADS1**	**FADS2**	**FADS3**	**ELOVL2**
Number of rare variants	15	20	15	248	108	247	94	322
Single-set *p*-value from simplified SKAT	0.659	***0.013***	0.529	0.081	0.388	0.255	0.403	***0.033***

*Bold denotes reaching statistical significance.*

In the individual gene-level results, considering alpha < 0.05, we find that two subsets, or genes (ELOVL2 and FFAR2), have raw *p*-values that reach statistical significance, with only borderline statistical evidence for FFAR4 (*p* = 0.081). Our hypothesis based on preexisting biological information was that FFAR4 would show a strong association with dichotomized obesity phenotype, but in fact just testing FFAR4 would not result in a significant finding (Table 3). In addition, neither testing the entire pathway as a single set nor testing the pathway as individual genes and applying a Bonferroni correction identifies this association. However, expanding to test the whole pathway using a multi-set approach does detect significant associations at the pathway level, allowing us to examine more closely the individual gene tests, identifying potentially novel associations between FFAR2 and ELOVL2 with obesity.

## Discussion

In this manuscript, we demonstrate that a multi-set testing (or “pathway-based”) approach shows similar or improved power when compared to single-set (or “gene-based”) methods. In particular, when the proportion of causal variants is similar across all sets, all methods (single-set and multi-set methods) performed similarly as the number of subsets increased, with the exception of the Bonferroni method, which suffers from the increased multiple testing penalty. In contrast, when the proportion of causal variants tends to be aggregated into certain subsets, multi-set approaches can lead to increases in statistical power with particularly large increases in power when the subsets containing causal variants have few variants overall.

### Uninformative vs. Informative Sub-Setting

The behavior illustrated in this manuscript suggests that the key to understanding the benefits of a multi-set testing approach is related to the *informativeness* of the sub-setting procedure. An uninformative sub-setting strategy will maintain equal proportions of causal variants across the subsets as was illustrated in *Scenarios #1* and *#2* of the simulations. Uninformative sub-setting is an approach which breaks down a larger set of variants into approximately “randomly selected” subsets of variants for testing. In this strategy, any causal variants in the set will be distributed approximately randomly across the subsets and, thus, the proportion of causal variants will be similar across the subsets, even if the size of some subsets is larger than others. As we demonstrated in simulation *Scenarios #1* and *#2*, multi-set testing strategies like the Fisher’s and SUMSTAT methods perform similarly to the single-set testing approach in this case, though with a modest decrease in power.

Informative sub-setting means that certain subsets contain higher proportions of causal variants than others. Thus, the subsets are helping to aggregate some of the true underlying signals in the data. Subsets are no longer “randomly” distributing variants to the sets. Multi-set approaches can significantly outperform single-set approaches in this situation by leveraging the additional statistical information provided by the sets being tested—even when there is no *a priori* knowledge of which subsets of variants are the ones containing the causal variants. In practice, this additional statistical information is likely to be contributed by using prior biological information to aggregate variants into subsets. As we demonstrated in simulation Scenarios #3 and #4, and the real data applications, multi-set testing strategies like the Fisher’s and SUMSTAT methods, and even the Bonferroni approach in some cases, outperform the single-set testing approach in this situation. These results are in line with prior work demonstrating that high proportions of causal variants in a set lead to high power (Derkach et al., 2014).

Given this insight, then, in practice, the choice of biologically informative subsets is paramount. Further research is needed to explore alternative subset approaches (e.g., genes, exons/introns, functional similarity, window based) and identify subset approaches that generate optimally sized and informative subsets for multi-set approaches. To date the nearly exclusive focus on gene-based tests is likely limiting our ability to elucidate the genetic architecture of complex diseases.

### Bonferroni and Multi-Set Testing Penalties

The conservative nature of the Bonferroni approach to multiple testing is well-known. Our inclusion of this approach was not simply to confirm these well-known results. Instead, the inclusion of the Bonferroni approach was to illustrate that other approaches to multi-set testing (e.g., Fisher’s and SUMSTAT), which appropriately control for multiple testing, have relatively little downside compared to a single-set approach when sub-setting is uninformative and large gains when subsets are informative. However, it is important to note that both the Fisher’s and SUMSTAT approaches do have some loss of power as compared to a single-set approach in the uninformative case. Thus, Fisher’s and SUMSTAT have an improved method of multiple testing penalty as compared to Bonferroni, but there is still a penalty for using them vs. a single-set approach when subsets are uninformative.

### Limitations

While our simulation settings do not consider all possible situations, they systematically (via the 2 × 2 factorial approach) provide an intuitive sense of the behavior of certain classes of tests in illustrative situations considering two important factors related to multi-set test power. Similarly, we acknowledge numerous other choices of test statistics (e.g., gsSKAT, GSEA, etc.) which may have advantages in certain practical situations (e.g., correlated genes). Furthermore, complexities of genetic data (e.g., multilevel sets; widely varying set sizes; linkage disequilibrium; variant weighting; related individuals) and genome-wide significance levels were not considered here. Our work to date provides a firm, intuitive foundation on which to build more complex investigations and real data applications, now having a basis on which to predict test behavior. Thus, while further investigations across a wide variety of additional scenarios are needed, and certain scenarios may gain particularly from particular choices of statistics, our investigation provides a broad and intuitive framework with which to anticipate test behavior, as is illustrated in our real data application. Furthermore, emerging approaches like a parametric bootstrap to assess genome-wide significance (Lin, 2019), among others (Povysil et al., 2019), are outside the scope of this manuscript. Finally, we considered large numbers of subsets of variants; however, exploring even larger numbers of subsets may be necessary to explore whether there is a limit to the patterns we observed in these most extreme cases.

### Conclusion

As the number of variants that are regularly identified and annotated as part of standard tests of genetic association increases, little is known about the behavior of standard “gene-based” (single-set) statistics in these settings. Our results suggest that there may be little downside and potentially a large upside (statistically improved power) to multi-set testing strategies even outside of traditional “pathway” based testing. Thus, considering subsets consisting of gene “subparts” (e.g., exons and introns; gene windows, etc.) may improve statistical power to elucidate disease etiology. However, future research is necessary in order to identify optimally informative ways to define subsets which provide maximal statistical power across a wide range of disease.

## Data Availability Statement

Publicly available datasets were analyzed in this study. This data can be found here: https://www.ncbi.nlm.nih.gov/gap/, with the dbGaP Study Accession Number of phs000007.v29.p10.

## Ethics Statement

The studies involving human participants were reviewed and approved by Dordt University IRB. The patients/participants provided their written informed consent to participate in this study.

## Author Contributions

RF, JB, and KL contributed to design and execution of simulations. RF, NT, and JW contributed to writing the manuscript. RF performed real data analysis. JW and NT contributed to design of project. All authors approved the final manuscript.

## Conflict of Interest

The authors declare that the research was conducted in the absence of any commercial or financial relationships that could be construed as a potential conflict of interest.
